# A história do ensino no Instituto Fernandes Figueira: entrevista com Susana Wuillaume

**DOI:** 10.1590/S0104-59702024000100005

**Published:** 2024-04-05

**Authors:** Susana Maciel Wuillaume, Saint Clair dos Santos Gomes

**Affiliations:** i Pesquisadora, Instituto Nacional de Saúde da Mulher, da Criança e do Adolescente Fernandes Figueira/Fiocruz.Rio de Janeiro – RJ – Brasil maleic@gmail.com; ii Coordenador da Vice-diretoria de Pesquisa e Inovação, Instituto Nacional de Saúde da Mulher, da Criança e do Adolescente Fernandes Figueira/Fiocruz. Rio de Janeiro – RJ – Brasil saint.junior@fiocruz.br

**Keywords:** History, Memory, Instituto Fernandes Figueira, História, Memória, Instituto Fernandes Figueira

## Abstract

Entrevista realizada com Susana Maciel Wuillaume, médica pediatra com larga experiência na docência e na gestão do Instituto Fernandes Figueira. São abordados diferentes temas da história institucional e da trajetória individual da entrevistada, como a organização da pós-graduação e dos programas de residências médica e a estruturação do próprio instituto. A entrevista faz parte de um projeto que documenta e investiga a história do Instituto Fernandes Figueira, que completa seu centenário em 2024.

## Abstract (en)

This interview was conducted with Susana Maciel Wuillaume, a pediatrician with broad experience in education and in the management of the Fernandes Figueira Institute. Various topics in the institution’s history and Wuillaume’s individual trajectory were covered, such as the organization of the graduate course and the medical residency programs and the structure of the institution itself. This interview is part of a project to document and investigate the history of the Fernandes Figueira Institute, which celebrates its one hundredth anniversary in 2024.

A presente entrevista foi realizada com Susana Maciel Wuillaume (1948- ), médica pediatra com grande experiência na docência e na gestão do Instituto Nacional de Saúde da Mulher, da Criança e do Adolescente Fernandes Figueira (IFF). Graduada pela Universidade Federal do Rio Grande do Sul, em 1973, Susana possui larga trajetória no campo da educação em saúde, sendo membro da Câmara Técnica da Comissão Nacional de Residência Médica do Ministério da Educação. É também presidente da Comissão Estadual de Residência Médica do Rio de Janeiro e ocupou, entre 2002 e 2009, o cargo de vice-diretora de Ensino do IFF.

Ao longo de sua carreira como pesquisadora, médica e docente, Susana abordou diferentes temas e problemas relativos ao campo da saúde materno-infantil, entre eles a implementação de diretrizes clínicas em unidades de tratamento intensivo neonatais, a atuação dos preceptores na residência médica em pediatria e o aleitamento materno. Esse percurso extenso também remete a diferentes contextos no âmbito da organização do cuidado materno-infantil no país e das políticas específicas para esse campo.

A trajetória de mais de quatro décadas de Susana na instituição permite uma perspectiva valiosa sobre a estruturação do Fernandes Figueira, principalmente quanto à organização da pós-graduação, na qual é professora desde 2001, e dos programas de residência na instituição. Embora parta do ponto de vista de uma gestora, a entrevistada oferece diferentes percepções sobre as práticas de cuidado realizadas no IFF, sua posição política na Fundação Oswaldo Cruz, o papel de iniciativas específicas como o banco de leite na construção de uma identidade institucional, entre outros pontos. Uma colaboração importante de alguém que tem participado de quase metade do período de existência do instituto e não só tem acompanhado processos de mudança, mas participado deles.

A entrevista foi realizada por pesquisadores do Instituto Fernandes Figueira, também com experiência na gestão do ensino e pesquisa no âmbito da pós-graduação. As perguntas foram condensadas, e os entrevistadores não estão especificados em cada questionamento, que foram realizados de forma alternada. Esta entrevista é parte de um projeto que documenta e investiga a história do Instituto Fernandes Figueira, que completa seu centenário em 2024.

*Susana, como você se aproximou da área da pediatria e chegou ao Instituto Fernandes Figueira? Quais eram as condições de trabalho e infraestrutura no IFF quando você ingressou?*

Eu me formei em medicina pela Universidade Federal do Rio Grande do Sul, em 1973, depois fiz um ano de residência nos EUA e um ano em Porto Alegre. Em 1977, ingressei no Instituto Fernandes Figueira como estagiária de pediatria em um ambulatório, tendo sido contratada em 1979. Depois fui chefe do Ambulatório Geral de Pediatria por um tempo, trabalhando lá até assumir cargo na direção do instituto, em 2002.

O hospital, naquela época, apresentava muitas fragilidades. Faltavam insumos, os equipamentos disponíveis eram antigos, havia problemas estruturais enormes. O ambulatório onde eu trabalhava funcionava por livre demanda, os pacientes iam chegando até 9:00h, faziam uma ficha para atendimento e esperavam nos bancos do corredor até serem chamados. A farmácia que existia era muito limitada. O funcionamento não era 24 horas, como hoje, e o cenário era totalmente diferente. Crianças apresentando sarampo ou desidratação grave eram frequentes, o que hoje não ocorre mais. Havia, ainda, uma movimentação na Fundação Oswaldo Cruz para que o terreno onde fica o IFF, na avenida Rui Barbosa, fosse vendido e o instituto fosse para o *campus* em Manguinhos. Em algum momento, porém, foram localizados documentos que diziam que o terreno havia sido doado especificamente para a criação de um hospital infantil; portanto, não seria possível vendê-lo, porque, se isso fosse feito, o dinheiro teria que voltar para a família doadora. Quando o Paulo Roberto Boechat^1^ assumiu a direção do Fernandes Figueira, ele iniciou um movimento para uma ampla reforma do hospital, por meio de articulação com Sergio Arouca,^2^ então presidente da Fiocruz. Com essa reforma, o instituto se transformou.

*Você tem uma trajetória importante na pesquisa e no ensino no IFF. Pode comentar um pouco sobre esse processo de criação e desenvolvimento da pós-graduação no instituto?*

Eu fui da segunda turma de mestrado do Fernandes Figueira, entrando em 1989^3^ e me formando em 1991. Na época, a formação pós-graduada era somente em pediatria, com uma turma de dez estudantes, todos médicos. É importante observar que a criação da pós-graduação no IFF teve muito a ver com a definição de um perfil de pesquisa para o instituto, uma forma também de romper com a ideia de que ali era “somente um hospital”. O Arouca e o Marco Antônio Barbieri^4^ foram muito importantes na articulação para a criação da pós-graduação no IFF, fortalecendo essa dimensão na instituição. Desde aquela época, já éramos uma das unidades com maior orçamento na Fiocruz, e ter uma área de ensino e pesquisa forte também ajudava na justificativa para maiores repasses de verba, tema sempre objeto de tensões na dinâmica institucional.

Eu tenho ótimas lembranças do tempo do mestrado. Éramos uma turma pequena, os professores eram excelentes, e a carga horária bastante extensa, muito maior do que atualmente. Muitos professores eram de fora da instituição. Lembro de ficarmos todos ao redor de uma mesa ampla, que até hoje é utilizada no instituto, na direção de ensino. Após concluir o mestrado, retornei ao trabalho exclusivo no ambulatório. Quando foi aberta a primeira turma de doutorado, eu já havia ingressado no programa do Instituto de Puericultura e Pediatria Martagão Gesteira, da Universidade Federal do Rio de Janeiro, o qual, porém, ainda estava em vias de credenciamento. Assim sendo, consegui transferência para o doutorado aqui do IFF, terminando em 2000.

Logo em seguida, tornei-me professora do programa de pós-graduação, ministrando uma disciplina sobre escrita de projetos de pesquisa. Em 2003, criei outra disciplina eletiva, denominada Processos Pedagógicos, que sempre lotava de inscritos, embora as pessoas não soubessem bem, de saída, do que tratava o curso. Eu queria que elas soubessem o que era aprender; o que era ensinar e que isso era um processo; queria discutir avaliação, que avaliação não é dar nota, a questão de uma avaliação formativa, de acompanhamento etc. E eu dizia para elas na primeira aula, que se saíssem do curso entendendo que não iriam passar conhecimento para ninguém, e sim ajudar as pessoas a adquirir o conhecimento, cada um de um jeito, meu objetivo teria sido alcançado. Então era discutido o processo de ensino e aprendizagem, tanto com aulas teóricas quanto com momentos de dramatização e atividades práticas.

Eu acho que o perfil da pós-graduação hoje é bastante diferente do que encontrei quando cursei ou mesmo quando comecei a lecionar. Um dos pontos principais é a mudança no perfil do aluno: inicialmente, a maioria dos estudantes de “pós” do IFF era de médicos, em seguida ingressaram profissionais de enfermagem, fisioterapia e serviço social, mas com predominância da medicina. Hoje, temos um perfil mais próximo do multiprofissional, inclusive com jornalistas cursando pós-graduação no instituto. Além da dimensão profissional, também houve uma transformação geracional, com ingressantes cada vez mais jovens. Eu sempre fazia um gráfico de “quem somos” na minha disciplina, que no início mostrava que o grupo consistia, sobretudo, de pessoas com mais tempo na instituição que decidiam se qualificar na pós-graduação. Com a mudança de perfil e o rejuvenescimento da turma, vieram também outras questões com implicações no ensino e aprendizagem, a exemplo da inclusão do celular na sala de aula, para a qual tive, inicialmente, grande resistência. A chegada da pandemia tornou isso tudo ainda mais intenso e complexo, devido à migração para o ensino remoto.

*Houve também uma mudança de perfil do programa da pediatria para saúde coletiva?*

Na verdade, ele estava na saúde coletiva desde o começo, tanto é que, em vez de ser um mestrado em pediatria, era em saúde da criança. E, sim, nós não aprendíamos como você trata uma úlcera, como você trata um “não sei o quê”, a gente falava sobre nutrição no Nordeste, a nutrição como um problema social etc. Havia muitas disciplinas, que se mantêm até hoje, com um perfil de discussão mais voltado para “saúde e sociedade”. Nós não estudávamos nenhuma patologia, então tinha mais esse enfoque social, por isso mesmo se chamava mestrado em saúde da criança, não em pediatria. Hoje existem muitos programas com esse perfil, mas na época éramos o único.

No período do mestrado, desenvolvi minha pesquisa com crianças em situação de rua no Centro do Rio de Janeiro. Consegui uma sala vazia na Fundação São Martinho, levei minha balança e atendi as crianças ali. Examinando, medindo e conversando com elas, para saber como era a vida que levavam, se iam à escola, como se alimentavam, onde moravam. Eu recebia respostas do tipo “ah, eu moro na marquise do Banco do Brasil”. Observava também as dinâmicas estabelecidas entre elas para viver naquele contexto, como quem pedia dinheiro e quem organizava os valores recebidos. Tinha gente que passava a semana toda na rua e no fim de semana ia para sua casa no subúrbio. Existia a casa, mas ficava muito caro o transporte da família para lá e para cá, então eles moravam durante a semana no largo da Carioca e voltavam para casa no fim de semana.

Foi possível observar que eles tinham uma altura menor do que as outras crianças de sua idade, mas eram mais bem nutridos do que as crianças que ficavam em casa na comunidade, aquelas que a mãe deixava em casa e ia trabalhar. Os que iam para a rua eram mais bem nutridos, porque eles pediam comida, e os que ficavam em casa dependiam daquele salário único que a mãe trazia para dividir para todo mundo.

*No doutorado você manteve esse perfil de pesquisa sobre as crianças em situação de rua?*

Não, no doutorado trabalhei com a residência médica em pediatria, com a preceptoria na residência. Comigo tinha acontecido como a tantos outros, um dia eu era residente, fui contratada como profissional, e no outro dia de manhã era preceptora. Então quis entender como funcionava essa dinâmica em um contexto mais amplo. Na época, não era um tema muito estudado na medicina, sendo abordado principalmente pela enfermagem.

*E quanto às residências, Susana, como se desenvolveram ao longo do seu tempo de Fernandes Figueira?*

A residência no Instituto Fernandes Figueira teve início quando Luiz Torres Barbosa, um dos pioneiros da pediatria no Rio de Janeiro, retornou dos EUA e abriu uma residência aqui, uma das primeiras em pediatria no país. Sendo que o Torres Barbosa ficou pouco tempo no IFF e migrou para o Hospital dos Servidores do Estado, levando com ele a residência. Temos na secretaria acadêmica fichas de matrícula de residentes desde 1961. No início, o ingresso deles ocorria por indicação, normalmente mobilizada pelo Fernando Olinto,^5^ com seus contatos em outros estados do país.

Quando entrei, havia três residências, em pediatria, obstetrícia e cirurgia pediátrica; também era ofertada uma especialização em genética. Por desconhecimento ou mesmo por hábito, todo mundo era chamado de residente, não havia diferenciações, o que gera hoje embaraços administrativos. Por exemplo, hoje a secretaria depara-se com um certificado de “residência em pneumologia no Fernandes Figueira”, mas de um período em que não tínhamos ainda o credenciamento para residência nessa área. Com a criação da Comissão Nacional de Residência Médica (CNRM),^6^ em 1977, o cenário mudou, havendo necessidade de inscrição e a realização de vistoria nas instituições – a nossa primeira ocorreu em 1978. Essa comissão apresenta a legislação com os requisitos mínimos para a existência de um programa de residência, o que também possibilitou a mobilização de pessoas e grupos para criação de novos programas, com bolsas pagas pelo próprio IFF.

A residência no Fernandes Figueira era considerada uma residência boa e foi aos poucos sendo aprimorada, sempre havendo grande procura pelos programas. Não costumam sobrar vagas, sempre tem muito mais candidatos do que vagas. Em 2023, houve um quadro geral de baixa procura em algumas áreas, como a neonatologia, e que acabou afetando a procura pelos programas daqui também, mas não é um fenômeno exclusivo do IFF.

*Você descreveu um cenário muito precário quando ingressou no instituto. Como era a situação quando você entrou para a direção?*

Em 2002, assumi o cargo de vice-diretora de Ensino do IFF. Nesse período, já havia acontecido a grande obra à qual me referi, realizada pelo Paulo Boechat, e a estrutura era a que temos atualmente. A principal questão envolvia a definição das funções e dos objetivos do hospital, qual o papel dele no sistema e na rede de saúde, se funcionava como centro de referência ou serviço geral por livre demanda. Por um tempo, atendíamos a quem chegasse no instituto, depois passamos a atender somente sob encaminhamento. Posteriormente, foi adotado o Sistema de Regulação (Sisreg).^7^

Tivemos épocas muito complicadas no ambulatório pediátrico, porque, de repente, o hospital mudou e mudou também o perfil do atendimento, quando veio a neurocirurgia pediátrica. A neurocirurgia veio, e é excelente, só que com ela vieram inúmeras crianças registradas naquele ambulatório específico e que, quando tinham algum problema mais geral, como uma dor de barriga, desciam para o ambulatório geral, que lotava. Também houve uma mudança no perfil epidemiológico das crianças atendidas, com mais ocorrência de doenças genéticas, de condições com cronicidade mais longa e complexa.

*Embora possua várias áreas fortes, o IFF é comumente reconhecido pela maternidade e pelo banco de leite. O que explica, na sua visão, essa característica?*

Isso é curioso, porque se você indica a um taxista que está vindo para cá, provavelmente escutará “ah, é lá na maternidade”. Nesse caso específico, essa identificação acontece porque as mães não pegam um táxi para levar as crianças para consultar, elas vão de ônibus. Mas, quando está nascendo a criança, elas vão de táxi, então, entre taxistas, predomina a identidade da maternidade. Nós fornecemos o atendimento emergencial e fazemos um acompanhamento detalhado após o parto, algo muito bem feito pela maternidade, o que também reforça essa imagem entre a população.

Mas esse perfil também passa por mudanças e acompanha as transformações do próprio hospital ao longo do tempo. Em tempos mais recentes, tivemos uma mudança de perfil com a maior incidência de pacientes crônicos, o que criou diversas demandas novas para o funcionamento do hospital, a exemplo da criação da residência multiprofissional. Esse novo cenário também demandou que o IFF fizesse o acompanhamento dos casos, pois outros serviços da rede de saúde não aceitavam casos complexos e crônicos, até mesmo por falta de infraestrutura. Em certa medida, essas mudanças no cotidiano hospitalar também afetaram a forma como as pessoas identificam o instituto.

*Quando você ingressou no instituto já funcionava o banco de leite?*

Funcionava, sim. Era muito curioso, porque quem o mantinha era praticamente a doutora Maria Rita Galloti, que morava no prédio ao lado aqui do instituto. E o banco de leite não era nada daquilo que é hoje. A doutora Maria Rita servia almoço para as mães que vinham doar leite, eram almoços maravilhosos! Ela também abria cadernetas de poupança para os bebês que acompanhavam as mães doadoras, tudo a partir de recursos dela, até onde eu sei. De vez em quando a gente falava no ambulatório: “está na hora do lanchinho da Maria Rita”, porque era um evento aberto a todas as pessoas. O leite era bem armazenado, com todos os cuidados, mas de uma forma muito mais simples do que é feito hoje. Quando entrou o João Aprígio,^8^ o banco foi transformado, com mais controle de qualidade e outros procedimentos para doação do leite (Almeida, 1999).

*E nessa construção de um perfil do hospital, como se desenvolveram as áreas mais específicas, como a genética?*

Quando eu ingressei, o setor de genética, na época comandado pelo José Carlos Cabral de Almeida, era bem conhecido. Gradativamente, a área foi crescendo, principalmente por meio da pesquisa e do programa de residência; o setor atraiu muita gente voltada para a pesquisa nessa área, desenvolvendo estudos e práticas de ponta. Além disso, o IFF começou a oferecer tratamentos para doenças raras, que não estavam disponíveis em outras instituições, como a *Osteogenesis imperfecta*. Com a criação das políticas nacionais de genética clínica e de doenças raras^9^ e a transformação do Fernandes Figueira em instituto nacional, passamos a ocupar o papel de centro de referência em genética clínica. Embora os últimos anos tenham sido difíceis, com a gestão pública e com a pandemia de covid-19, não vi falha nos serviços por falta de recursos, parece ainda um setor bem forte.

*Existe uma interpretação de que a emergência da zika teve um papel importante na trajetória do IFF. Como você observa esse processo?*

Eu concordo, a zika foi um fenômeno muito importante para a instituição. Primeiramente, houve um grande receio sobre como faríamos na parte clínica, no atendimento das crianças e das mães, algo que rapidamente foi organizado. Então, vieram diferentes fontes de financiamento para as pesquisas sobre o tema, incluindo recursos internacionais, como da Fundação Bill e Melinda Gates, o que levou a uma ampliação do nosso alcance em termos de repercussão de pesquisa e de reconhecimento institucional. Porém, tudo isso só foi possível porque já havia uma estrutura de cuidado e pesquisa bem desenvolvida, com recursos humanos qualificados e prontos para o desafio de lidar com uma nova doença e uma grande emergência sanitária internacional.

O grande volume de recursos permitiu, entre outras coisas, que nos equipássemos ainda mais, com desenvolvimento importante nos campos da medicina fetal e seguimento neonatal. Este último ocupava uma pequena sala e passou a receber equipamentos de ponta, como o Pea Pod, uma máquina que faz composição corporal por descompressão de ar; só o IFF e um hospital de São Paulo possuem tal tecnologia. Então, acho que houve uma sinergia entre as mobilizações para fornecer respostas à emergência da zika e uma estrutura institucional forte do instituto. Certamente, foi um ponto de inflexão, impactando, aliás, a posição do IFF dentro da Fiocruz, nas já mencionadas disputas políticas; hoje em dia ocupamos um lugar bem mais consolidado e com bastante visibilidade.

*Susana, temos falado muito sobre o perfil do Fernandes Figueira e das mudanças que aconteceram na instituição ao longo do seu tempo aqui. Quais elementos você considera fundamentais dessa trajetória institucional mais recente, no século XXI?*

Primeiramente, eu destacaria que o instituto efetivamente cresceu e se qualificou cada vez mais, em todas as áreas. Hoje, oferecemos um atendimento primoroso, com muita qualidade, não deixando nada a desejar em relação a grandes instituições privadas, que têm como diferencial seus serviços de hotelaria, com pouco a ver em termos do cuidado com a saúde. Ainda assim, acredito que a mudança para um perfil de pacientes com mais cronicidade coloca desafios importantes para o nosso trabalho aqui no hospital.

Outro aspecto fundamental diz respeito à humanização, algo que não era discutido quando ingressei no IFF. Hoje, estamos atentos a aspectos específicos do cuidado, a exemplo da acessibilidade e do cuidado com a saúde mental, havendo estruturas institucionais específicas para lidar com essas dimensões do cuidado. Oferecemos medicamentos, equipamentos e serviços de alta complexidade, atuamos como centro de referência em diferentes áreas do cuidado da criança e da mulher. Uma das coisas que eu realmente admiro no Fernandes Figueira é que existe um cuidado com o paciente muito grande em todos os setores! Há uma preocupação muito grande com o paciente, com a família do paciente, com a saída do paciente, como é que vai ser depois que ele sair do hospital.

*E o que podemos esperar do Instituto Fernandes Figueira nos próximos anos?*

Eu espero que sejam bons anos. Nesta nova conjuntura eu não sei o que esperar, mas eu acho que o governo Lula, ainda mais tendo a Nísia Trindade no Ministério da Saúde, tem chance de querer fazer uma série de coisas interessantes! Porém, existem pressões diversas sobre o governo, diferentes elementos políticos que precisam ser balanceados. Tenho segurança de que Nísia conhece bem o IFF, e com ela conseguiremos atuar como um instituto nacional, coordenando estratégias e políticas no âmbito da saúde da criança e da mulher, seguindo a nossa tradição de instituição de assistência, ensino e pesquisa. O risco reside na capacidade que determinados grupos de interesse terão em encaminhar agendas mais machistas ou em reduzir o papel de políticas mais progressistas no âmbito da saúde, mas existem esperanças e boas expectativas para os próximos anos, espero que se confirmem.

## NOTAS

1 Paulo Roberto Boechat é formado em medicina pela Universidade Federal Fluminense, com residência em cirurgia pediátrica no Instituto Fernandes Figueira e na Universidade de Miami. Foi coordenador do Departamento de Cirurgia Pediátrica de 1980 a 1985, quando foi eleito diretor do IFF, até 1993. De 1994 até 2013, ano de sua aposentadoria, foi coordenador do Departamento de Cirurgia Pediátrica do IFF.

2 Sergio Arouca, médico e sanitarista, foi professor da Escola Nacional de Saúde Pública. Como presidente da Fiocruz, foi o responsável pela reintegração dos dez cientistas cassados pela ditadura militar. Ocupou a presidência da instituição até abril de 1988, quando se exonerou para concorrer à vice-presidência da República na chapa do Partido Comunista Brasileiro, com Roberto Freire.

3 O Programa de Pós-graduação em Saúde da Criança e da Mulher foi criado em 1988. O eixo temático do IFF/Fiocruz corresponde a um importante marco de atuação e foco, tanto na área de assistência, gestão e formação de docentes e de pesquisadores quanto na emergente construção de tecnologias, práticas e saberes direcionados aos grupos em situação de vulnerabilidade.

4 Marco Antonio Barbieri é professor titular de pediatria e, desde 1988, coordenador do Núcleo de Estudos da Saúde da Criança e do Adolescente da Faculdade de Medicina de Ribeirão Preto, da Universidade de São Paulo. Na mesma instituição estudou medicina (formado em 1967) e foi médico residente em pediatria (1968-1969). Disponível em: http://lattes.cnpq.br/0765555733544821. Acesso em: 11 set. 2023.

5 Fernando Olinto formou-se em 1956, pela Faculdade de Ciências Médicas da Universidade do Estado do Rio de Janeiro, e realizou residência em pediatria no Instituto Fernandes Figueira. No IFF, presidiu a Comissão Diretora do Curso de Pediatria e representou a instituição em diferentes fóruns acadêmicos. Em 1983, presidiu a Comissão Organizadora do Simpósio Internacional de Pediatria, realizado no Rio de Janeiro.

6 Criada pelo decreto n.7.562/2011, de 15 de setembro de 2011, a CNRM exerce função de regulação, supervisão e avaliação de instituições que ofertam residência médica e de programas de residência médica.

7 O Sisreg é um programa desenvolvido no âmbito do Datasus, disponibilizado gratuitamente para estados e municípios e destinado à gestão de todo o Complexo Regulador, desde a rede de atenção primária até a atenção especializada, visando regular o acesso aos serviços de saúde do Sistema Único de Saúde e potencializar a eficiência no uso dos recursos assistenciais.

8 João Aprígio Guerra de Almeida é graduado em engenharia de alimentos pela Universidade Federal de Viçosa (UFV, 1981), mestre em microbiologia (UFV, 1986) e doutor em saúde da mulher e da criança (Fiocruz/IFF, 1998). Fundador e coordenador, de 1987 a 2020, da Rede Brasileira de Bancos de Leite Humano; chefe do Centro de Referência para Bancos de Leite Humano do IFF. Disponível em: http://lattes.cnpq.br/9193103361359168. Acesso em: 11 set. 2023.

9 Em 2009, foi instituída a Política Nacional de Atenção Integral em Genética Clínica, que deu ênfase ao aconselhamento genético e às ações no sentido do cuidado integral. Em 2014, o Ministério da Saúde instituiu a Política Nacional de Atenção Integral às Pessoas com Doenças Raras, que teve como destaque a implantação dos Serviços de Atenção Especializada em Doenças Raras. Ver Araújo Neto e Teixeira (2020) e Vieira (2019).
